# Decreased lymph node estrogen levels cause nonremitting progressive experimental autoimmune encephalomyelitis disease

**DOI:** 10.1093/pnasnexus/pgaf010

**Published:** 2025-01-20

**Authors:** Shehata Anwar, Po-Ching Patrick Lin, Lazaro Pacheco, Kazuhiro Imai, Zhengzhong Tan, Ziyuan Song, Yuki Wakamatsu, Yoshihiro Minamiya, Jianjun Cheng, CheMyong Ko, Makoto Inoue

**Affiliations:** Department of Comparative Biosciences, The University of Illinois at Urbana-Champaign, 2001 South Lincoln Avenue, Urbana, IL 61802, USA; Faculty of Veterinary Medicine, Department of Pathology, Beni-Suef University (BSU), Beni-Suef 62511, Egypt; Department of Comparative Biosciences, The University of Illinois at Urbana-Champaign, 2001 South Lincoln Avenue, Urbana, IL 61802, USA; Materials Science and Engineering, University of Illinois at Urbana-Champaign, 1304 West Green Street, Urbana, IL 61801, USA; Department of Thoracic Surgery, Akita University Graduate School of Medicine, 1-1-1 Hondo, Akita 010-8543, Japan; Materials Science and Engineering, University of Illinois at Urbana-Champaign, 1304 West Green Street, Urbana, IL 61801, USA; Materials Science and Engineering, University of Illinois at Urbana-Champaign, 1304 West Green Street, Urbana, IL 61801, USA; Department of Thoracic Surgery, Akita University Graduate School of Medicine, 1-1-1 Hondo, Akita 010-8543, Japan; Department of Thoracic Surgery, Akita University Graduate School of Medicine, 1-1-1 Hondo, Akita 010-8543, Japan; Materials Science and Engineering, University of Illinois at Urbana-Champaign, 1304 West Green Street, Urbana, IL 61801, USA; School of Engineering, Westlake University, Hangzhou 310030, China; Department of Comparative Biosciences, The University of Illinois at Urbana-Champaign, 2001 South Lincoln Avenue, Urbana, IL 61802, USA; Department of Comparative Biosciences, The University of Illinois at Urbana-Champaign, 2001 South Lincoln Avenue, Urbana, IL 61802, USA

**Keywords:** lymph node, estrogen, aromatase, experimental autoimmune encephalomyelitis, multiple sclerosis

## Abstract

Estrogen, a steroid hormone synthesized by both gonadal and nongonadal tissues, plays a pivotal role in modulating immune responses, including reducing relapse rates in relapsing–remitting multiple sclerosis (MS). This study explored the expression of aromatase, the enzyme responsible for estrogen synthesis, in lymph nodes (LNs) and its potential role in the pathogenesis of MS using a mouse model. We utilized Cyp19-RFP mice where cells that express or have previously expressed the Cyp19 gene (encoding aromatase) are marked by red fluorescent protein (RFP). RFP was detected in the high endothelial venules of all morphologically identifiable LNs, indicating aromatase activity within these tissues. We discovered that LNs actively synthesize 17β-estradiol, but this activity declines with age. Targeted delivery of an aromatase inhibitor specifically to LNs induced an interferon-β-resistant experimental autoimmune encephalomyelitis (EAE) phenotype. This phenotype was accompanied by significant gray matter atrophy in the spinal cord. These findings underscore LNs as crucial sites of de novo 17β-estradiol production, potentially contributing to nonremitting EAE phenotypes. The observed decline in 17β-estradiol likely exacerbates MS pathogenesis in aging mice. Importantly, aromatase expression in human cervical LNs suggests that these sites may similarly contribute to estrogen synthesis in humans, potentially opening new avenues for understanding and treating MS.

**Significance Statement**

Multiple sclerosis (MS) is a debilitating neurodegenerative disease with limited treatment options. Our study reveals that lymph nodes (LNs) are key sites for 17β-estradiol synthesis, which declines with age, driving nonremitting experimental autoimmune encephalomyelitis progression. Targeting estrogen pathways within LNs offers a promising new therapeutic strategy for progressive MS.

## Introduction

Multiple sclerosis (MS) is a chronic autoimmune and neurodegenerative disease of the central nervous system (CNS) affecting nearly 1 million individuals in the United States and almost three million people worldwide (1–3). MS is characterized by demyelination, neuronal degeneration, and loss of neurons, with profound heterogeneity in disease presentation, trajectory, and treatment efficacy (2, 4). It is the most prevalent neurological condition that affects young adults, hits during the years when productivity is at its highest, and has a lasting detrimental impact on the patients, their families, and the healthcare system (5, 6).

The prevalence of MS is higher among females than males (7, 8). Nevertheless, men are more likely to be diagnosed with primary progressive MS with a poor prognosis (9, 10). Underlying hormonal differences may account for the sex-specific ratios and the severity of MS (11). Elevated estrogen levels during pregnancy promote immune tolerance and exert neuroprotective effects (12). Large-scale human studies revealed that estrogens significantly influence MS pathology with favorable prognosis. For example, MS relapse rates decrease as pregnancy progresses, a period marked by a steady rise in circulating estrogen (13–16). Likewise, postmenopausal women with MS frequently experience an increase in disease flare-ups, which coincides with a decline in estrogen levels (15, 17). Similarly, several clinical studies have shown the beneficial impact of estrogen on patients with MS (18, 19). The inverse relationship between endogenous estrogen and disease severity is recapitulated in the well-established MS murine model named experimental autoimmune encephalomyelitis (EAE). For instance, ovariectomized or castrated mice and rats showed more severe EAE disease than the intact ones (20–23). Moreover, 17β-estradiol treatment reduced the clinical manifestations of EAE disease. Recently, we have investigated the functional role of estrogen and estrogen receptor alpha (ERα) on EAE disease phenotypes and how endogenous estrogen/ERα signaling contributes to changes in EAE pathogenesis and phenotype (24). We found that estrogen synthesized by nongonadal tissues, but not gonadal estrogen, is crucial for regulating EAE phenotypes. However, the nongonadal tissues producing estrogen for such function have yet to be fully identified.

Aromatase, the enzyme encoded by the *Cyp19* gene, is the key enzyme for estrogen biosynthesis. It converts androstenedione and testosterone to estrone and 17β-estradiol, respectively. The current study aimed to determine whether aromatase is expressed in lymph nodes (LNs) irrespective of their location. Additionally, it investigated whether aromatase expression contributes to MS heterogeneity in the widely used EAE model.

## Results

### Lymph nodes in both mice and humans express aromatase

**Fig. 1.**
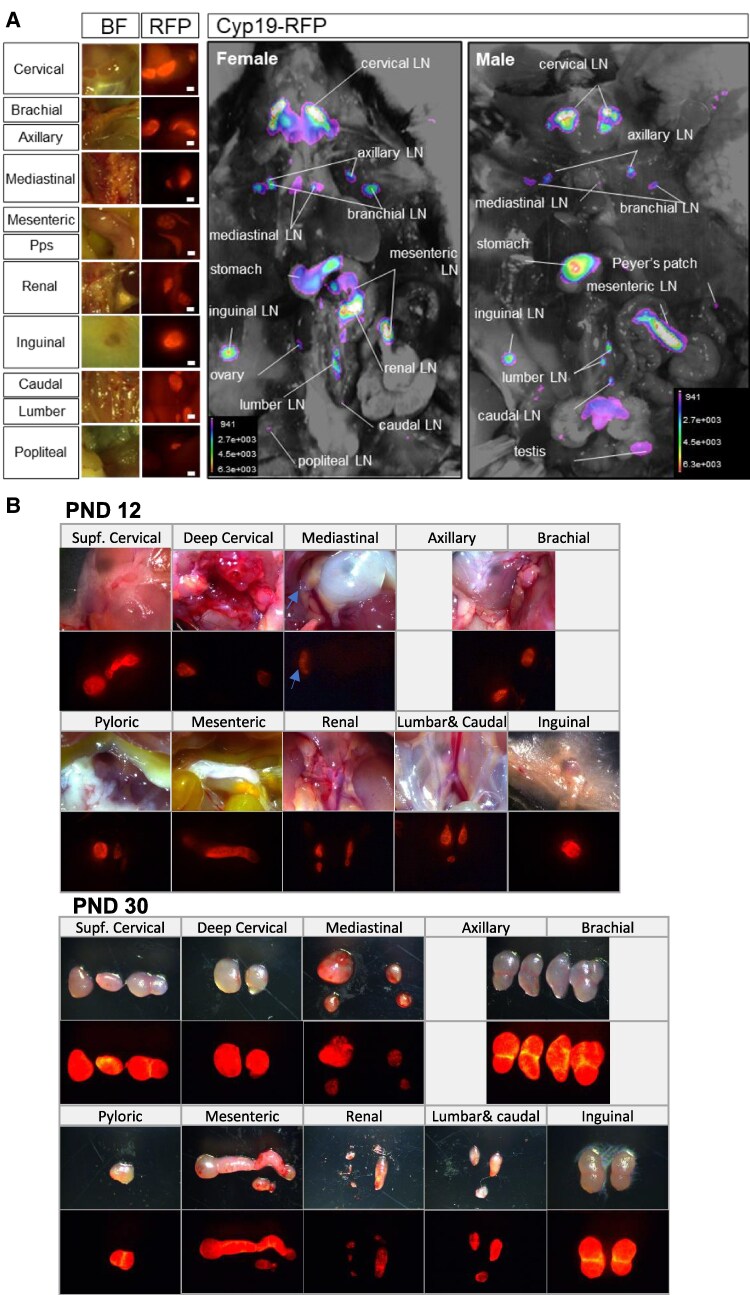
A representative image of Cyp19 promoter-derived RFP expression. A) Representative RFP signals in cervical, brachial, axillary, mediastinal, mesenteric, renal, inguinal, caudal, lumber, popliteal LNs, and Peyer’s patches of 8-month-old female and male mice. B) Representative RFP signal in superficial and deep cervical, mediastinal, axillary, brachial, pyloric, mesenteric, renal, lumbar, caudal, and inguinal LNs of mice PNDs 12 and 30.

**Fig. 2.**
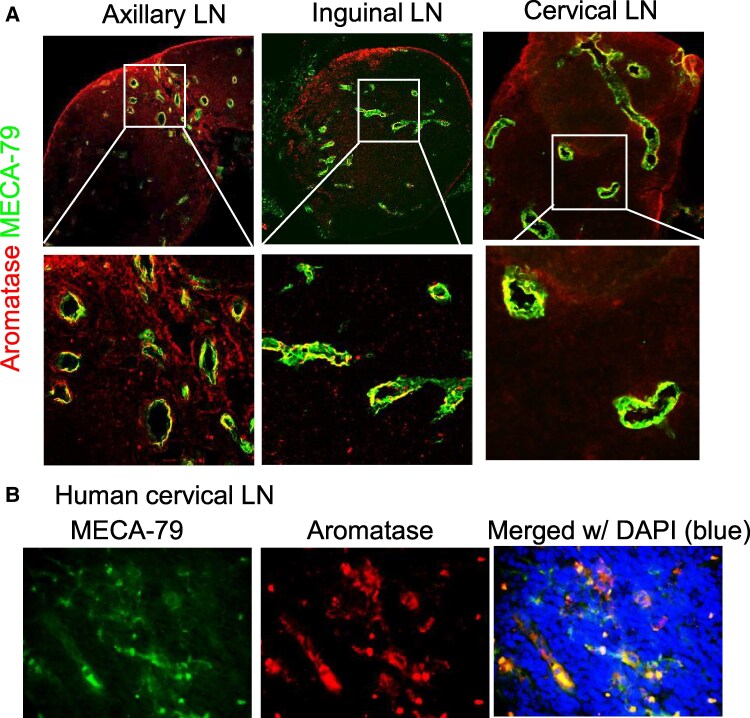
Immunohistochemical expression and localization of aromatase in mouse- and human-derived LNs. A) Representative photomicrographs of aromatase and MEC-79, a marker of HEVs in cervical, axillary, and inguinal LNs of an 8-month-old mouse. B) Representative photomicrographs of aromatase and MEC-79 in a human cervical LN.

To identify potential nongonadal tissues producing estrogen, we developed a transgenic mouse line with red fluorescent protein (RFP) expression in Cyp19-expressing cell lineages (25). In this mouse (Cyp19-RFP), cells that express or have previously expressed the *Cyp19* gene (encoding aromatase) would be identified by the RFP expression. This mouse, Cyp19-RFP, was generated by cross-breeding the Cyp19-iCre mice with the Ai9 td-tomato reporter mouse line. In adult male and female mice, high-intensity RFP signals were detected in all LNs, including cervical, axillary, brachial, and inguinal LNs, which are implicated in MS pathogenesis (26, 27) and EAE (28, 29) pathogenesis (Fig. 1A). On postnatal day (PND) 12, RFP signal was detected in LNs (Fig. 1B), which include superficial and deep cervical, mediastinal, axillary, brachial, pyloric, mesenteric, renal, lumbar, caudal, and inguinal LNs (Fig. 1B). At PND 30, strong RFP signals continued to be observed in all the aforementioned LNs (Fig. 1B). Aromatase expression in LNs of adult mice was further confirmed by IHC (Fig. 2A). Aromatase expression was localized to high endothelial venules (HEVs), which were identified by an antibody against MECA-79 that recognizes sulfated L-selectin ligands of HEVs (29–31) (Fig. 2A). Similarly, aromatase expression was also detected in the HEVs of human cervical LNs (Fig. 2B). We previously reported that 17β-estradiol is highly expressed in LNs, and its expression is not affected by ovariectomy (24). Therefore, all these results suggest that LNs are sites for 17β-estradiol synthesis.

### Albumin-coated nanoparticles were developed for targeted delivery to LNs

**Fig. 3.**
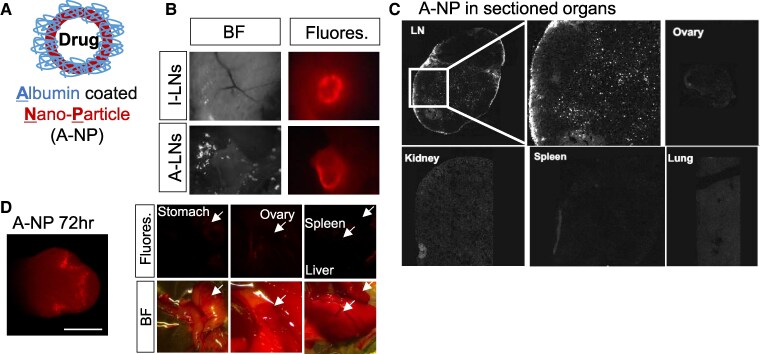
A-NPs distribution. A) Graphical design of A-NP. B) Representative bright-field and fluorescence images of inguinal (I-LNs) and axillary (A-LNs) LNs 6 h after s.c. injection of rhodamine-loaded A-NP. C) Representative rhodamine fluorescence (shown as white) images of 30-µm tissue sections 12 h post-rhodamine A-NP treatment. D) Representative image of rhodamine-fluorescence-loaded nanoparticles in inguinal LN 72 h post-injection.

Lymph nodes are key locations where T cells reactivate upon contact with dendritic cells in MS and its animal model of EAE (32). The potential significance of estrogen synthesized in LNs during EAE pathogenesis was assessed. We first engineered a nanoparticle drug delivery system specifically targeting LNs. As a natural biomacromolecule, albumin possesses characteristics of an ideal carrier for drug delivery, such as good water solubility, biodegradability, lack of toxicity, and minimal immunogenicity (33). Compounds that bind to albumin can be effectively transported to draining LNs, making albumin an ideal moiety for targeting LNs (34, 35). Leveraging the nature of albumin, albumin-coated nanoparticles (A-NPs; Fig. 3A) were synthesized as an LN-targeted drug delivery vehicle. To determine the LN specificity, we first made and tested rhodamine-loaded A-NPs. Upon subcutaneous (s.c.) injection of rhodamine-loaded A-NPs, fluorescence was observed in LNs, including the axillary and inguinal LNs 6 h after injection (Fig. 3B). In sectioned LNs, rhodamine fluorescence was also detected in both the outer parameter and parenchyma of LNs 24 h after injection (Fig. 3C). No off-targeting fluorescence signals were observed in other tissues, including the kidney, spleen, or ovary (Fig. 3C). These results confirm that A-NP is delivered to and retained in LNs with no significant off-targeting effect.

### Inhibition of aromatase in LNs causes prolonged interferon-β-resistant EAE

**Fig. 4.**
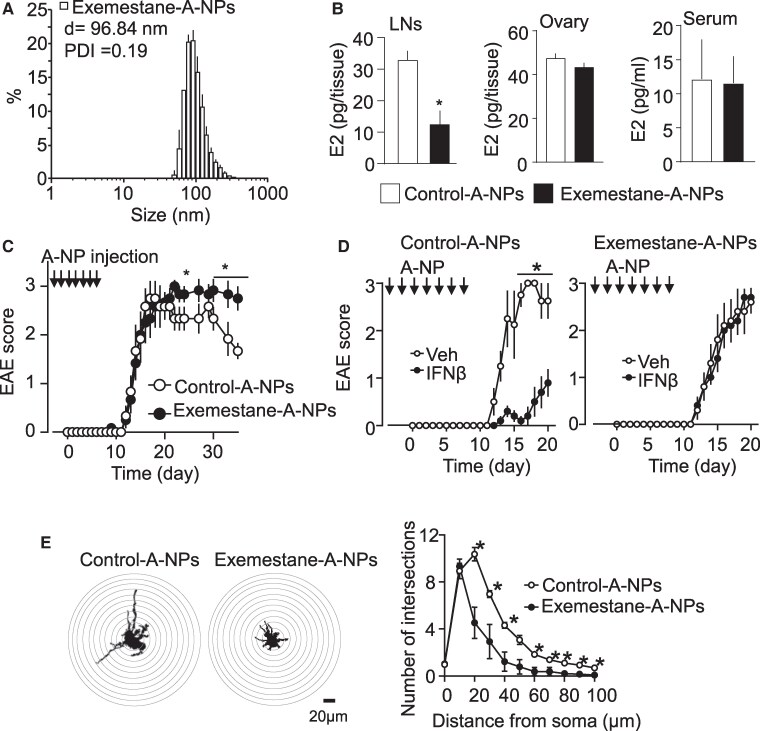
Inhibition of aromatase in the LNs causes IFN-β-resistant and CNS neurotoxic EAE. A) The diameter of exemestane-loaded a-NP was 96.84 nm with a low polydispersity index (< 0.19), which is within the optimal size range of nanoparticles for LN targeting. B) 17-Beta estradiol (E2) level in murine LNs, ovaries, and serum after day 4 of the s.c. injections of exemestane (1.5 µg/mouse)-A-NPs at days 0 and 2. *n* = 4–7 mice per group. C) EAE score in mice with vehicle- and exemestane-loaded A-NPs. Arrows indicate the A-NP injection day (−4, −2, 0, 2, 4, 6, and 8 days; day 0 is EAE induction day). *n* = 5. D) IFN-β sensitivity in EAE mice treated with vehicle- or exemestane-loaded A-NP. *n* = 5 mice per group. Arrows indicate the A-NP injection day (−4, −2, 0, 2, 4, 6, and 8 days; day 0 is EAE induction day). E) Neurite outgrowth of the neurons in Golgi-stained spinal sections of EAE mice treated with vehicle- or exemestane-loaded A-NP at 33 dpi quantified using the Sholl analysis method. Sholl rings are separated by 10 μm (*n* = 5 mice per group, 20 neurons analyzed per animal) with an end radius of 100 μm. Data are representative of two to three independent experiments. *P < 0.05.

To determine whether LN-synthesized estrogen plays a role in EAE pathogenesis, A-NPs were loaded with exemestane, an aromatase inhibitor (36), and used to selectively inhibit aromatase activity in the LNs. The diameter of exemestane-loaded A-NP was 96.84 nm with a low polydispersity index (< 0.19), which is within the optimal size range of nanoparticles for LN targeting (Fig. 4A). Briefly, with a diameter <10 nm, nanoparticles are mainly absorbed into the bloodstream, and with a diameter >100 nm, nanoparticles remain trapped in interstitial tissue (37, 38). As proof of A-NP design efficacy, we show that 17β-estradiol concentration in the LNs significantly decreased 4 days after two s.c. injections of exemestane-loaded A-NPs. As expected, treatment of exemestane-loaded A-NPs did not alter 17β-estradiol levels in the ovary, a major 17β-estradiol synthesizing tissue, and in the serum, which highly contains ovary-derived 17β-estradiol (Fig. 4B). Then, to examine whether inhibition of estrogen synthesis in the LNs is sufficient to induce phenotype alterations in EAE mice, C57BL6 mice were injected with exemestane-loaded A-NPs or placebo vehicle-loaded A-NPs (Control-A-NPs) before and after EAE induction. Placebo-treated EAE mice entered disease remission 21 days post-induction (dpi) (Fig. 4C). However, mice treated with exemestane-loaded A-NPs did not enter remission (Fig. 4C). Furthermore, while interferon-β (IFN-β), a relapsing–remitting MS drug, suppressed disease development in the vehicle-loaded A-NP-treated mice, mice treated with exemestane-loaded A-NP did not respond to IFN-β treatment (Fig. 4D).

Prolonged motor paralysis in response to EAE induction is associated with CNS neuronal damage in the spinal cord's ventral horn regions where lower motor neurons reside (39–41). Previously, we reported that IFN-β-resistant EAE mice show severe motor neuron damage (42). In Golgi-Cox neuronal staining (43), the morphology and connectivity of Golgi-Cox-stained neurons showed a significant difference between EAE mice that were injected with exemestane-loaded A-NPs and placebo as indicated by percentage area stained with Golgi-Cox neuronal staining. Sholl analysis (44, 45) showed a significant decrease in dendritic length and number of intersections from exemestane-loaded A-NP-treated mice when compared with control (Fig. 4E). These results suggest that estrogen levels in LNs affect EAE disease outcome.

### 17β-estradiol level declines in aging mice

**Fig. 5.**
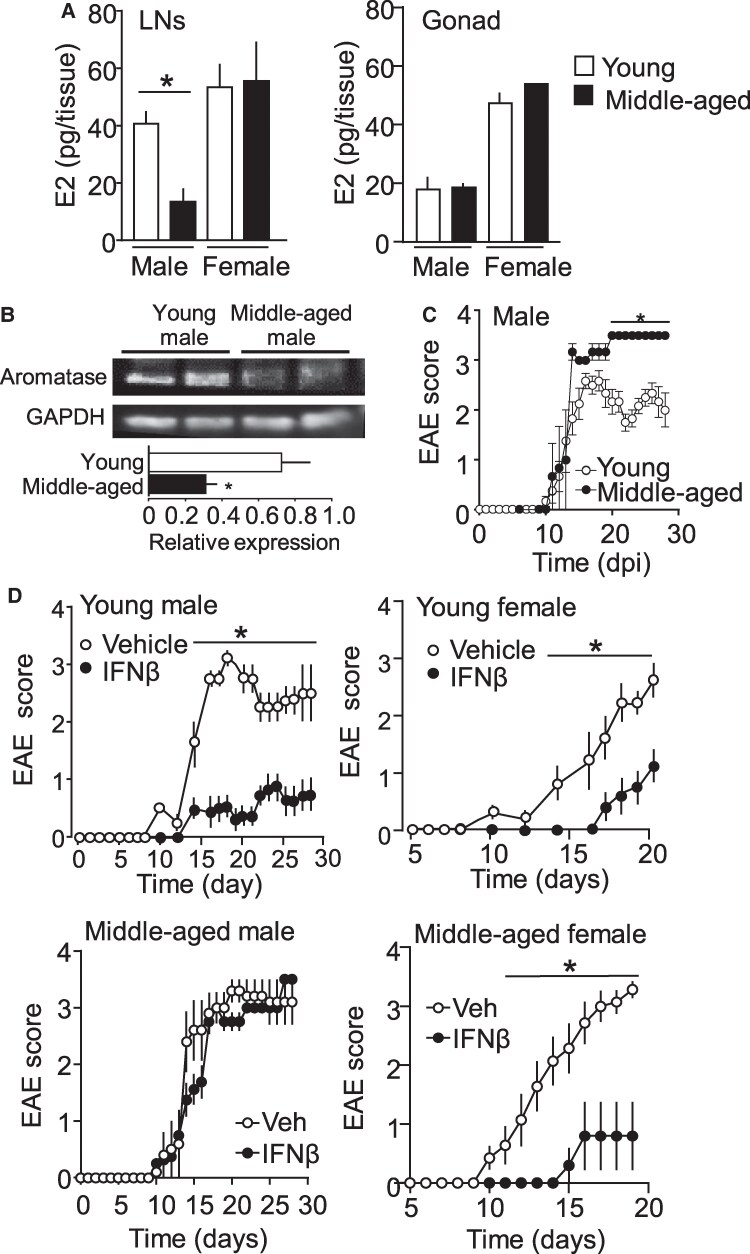
Aging elicits LN 17β-estradiol reduction and EAE phenotype alteration. A) The 17β-estradiol expression levels in the LNs and gonads of young-aged (2-month-old) and middle-aged (8-month-old) male and female mice. *n* = 5–11 mice per group. B) Semiquantitative analyses of aromatase expression by western blotting in the LNs of young- and middle-aged male mice. GAPDH was used as a loading control. *n* = 5. C) EAE diseases in young- and middle-aged male mice. *n* = 5, 6. D) IFN-β sensitivity in young- and middle-aged male and female mice. *n* = 5–8. *P < 0.05.

Aging is a factor in MS development and its pathogenicity (46). Interestingly, the 17β-estradiol contents in LNs of 8-month-old mice (middle-aged mice) are comparable to 38–40 years of age in humans (47), and 2-month-old mice (young mice) showed a sex-dependent pattern. Gonadal 17β-estradiol contents were not different between 2- and 8-month-old mice. Interestingly, 17β-estradiol contents in the LNs were significantly lower in the 8-month-old male mice but not in the female mice than those in the 2-month-old mice (Fig. 5A). In support, lower aromatase expression was seen in 8-month-old male mice, compared with 2-month-old male mice, as determined by western blotting analysis (Fig. 5B). Interestingly, 8-month-old male mice developed a nonremitting severe EAE phenotype (Fig. 5C), similar to EAE disease in ERα^−/−^ mice that we previously reported (24). While middle-aged female EAE mice were responsive to IFN-β treatment, middle-aged male EAE mice were IFN-β resistant (Fig. 5D). These results indicate that aging-driven reduction in LN's estrogen synthesis plays a key disease phenotype-altering role in EAE pathogenesis.

## Discussion

Estrogens are primarily produced in gonads. However, it is also well established that estrogens are synthesized in nongonadal tissues, producing estrogen, such as the liver and brain (48–50). Recently, we reported de novo 17β-estradiol synthesis in gut-associated lymphoid tissue, such as the mesenteric LNs and Peyer's patches (25). In the current study, we further examined estrogen synthesis in all LNs in mice, either superficial or deep, including pectoral axial and cervical LNs. Further, we report that human LNs express aromatase. Ovariectomy in mice does not alter LN's estrogen contents (51). Thus, we demonstrate that LNs are indeed sites of de novo estrogen synthesis and that estrogens produced in LNs are retained in their tissue microenvironment.

Notably, LNs maintain significantly higher 17β-estradiol concentrations than peripheral blood (25), which can be as high as gonads (25). Nongonadal synthesized estrogens are thought to act and be metabolized locally, which limits their systemic effects (52). Despite the small amount of estrogen produced in each tissue, local tissue estrogen concentrations could be sufficient to exert a biological impact locally (53). This highlights the potential impact of LN-derived estrogen on immune regulation. LNs are secondary lymphoid organs that regulate immune responses of the adaptive immune system. LNs are the control centers where the body starts immune responses. DCs bring antigens from external invaders to the LNs, searching for responsive T cells to present the antigens. Once activated, T cells divide rapidly to form a clone army that searches the body for the source of the invasion and removes it.

Several direct or indirect mechanisms have been proposed through which estrogen contributes to ameliorating MS severity, such as effects on autoreactive T cells and DC (24, 54) and modulation of myeloid cell functions (55). Estrogen enhances DC function in the innate immune system by up-regulating antigen presentation and co-stimulatory molecule expression, such as CD80 and CD86 via ERα. This enhancement improves the ability of DCs to activate T cells and initiate immune responses (48). Within the adaptive immune system, estrogen influences T cell activation and differentiation. Specifically, estrogen signaling promotes the expansion of regulatory T cells, mediated by increased interleukin-10 and transforming growth factor-β production. This shift toward an immunosuppressive profile helps maintain immune tolerance and suppress inflammatory pathways. Concurrently, estrogen modulates the balance between Th17 cells and Tregs, favoring the latter and mitigating autoimmune-driven inflammatory responses (56). Beyond its role in immune modulation, estrogen exhibits neuroprotective effects by reducing peripheral immune activation and dampening cytokine-driven neuroinflammation. By decreasing the infiltration of immune cells into the CNS, estrogen helps shield neurons from inflammatory damage. These protective effects have been demonstrated in EAE and other models of neuroinflammation, underscoring the therapeutic potential of estrogen-based interventions (48).

Despite evidence indicating that estrogen has disease-modifying impacts on patients with MS and the EAE model, most research has focused on studying estrogen's impact on MS severity. However, little is known regarding the effect of estrogen on MS phenotypes. Furthermore, most studies examined the impact of estrogen on EAE development using exogenous estrogen treatment (57). Appreciating the impact of endogenous estrogen on EAE pathogenesis has been challenging because treatment studies usually rely upon exogenous estrogen with doses that usually exceed endogenous estrogen levels. Because ER signaling is highly dependent on ligand concentration (58), supra-physiologically high levels of exogenous estrogen likely may not reflect the physiological actions of endogenous estrogen and its receptor functions. We have recently reported our results regarding the effect of endogenous estrogen and ERα on EAE disease phenotypes and how endogenous estrogen/ERα signaling contributes to changes in EAE pathogenesis and phenotype (24). Briefly, the absence of ERα signaling in dendritic cells increases endogenous IFN-β production via the lack of TRAF3 suppression, which has been clinically associated with blunted IFN-β response in patients with MS (24). Moreover, the absence of ERα signaling increases membrane lymphotoxin (mLT), thus triggering the development of an altered EAE disease pathway that is primarily driven by the mLT-LTβR signaling pathway, which cannot be suppressed by exogenous IFN-β (24). Importantly, LTβR signaling on CD4^+^ T cells up-regulates neurotoxic molecules, such as semaphorin 6B and ephrin B1, leading to CNS neuronal damage (24). Thus, these alternated signaling may contribute to altered EAE phenotype when LN's estrogen synthetic capacity changes. Because estrogen has neuroprotective effects (56, 59), it is still possible that LN-derived estrogen reaches the CNS and protects neurons in the CNS. In addition, the present report found that exemestane-A-NP-treated mice show IFN-β-resistant EAE disease. Previous reports suggest that IFN-β-resistant EAE mice show prolonged disease until at least day 60 (39). Thus, exemestane-loaded A-NP-treated EAE mice may also show more prolonged disease than day 33, which we evaluated in the present study. The next interesting question is whether all IFN-β-resistant EAE mice have a similar prolonged disease phenotype. Treatment of patients with estriol, an endogenous estrogen, alone (18, 60), or combined with an immune-modulatory drug (61) produces favorable therapeutic outcomes such as reduced lesion load (18) and relapse rate (61). However, while estrogen treatment produced promising outcomes, clinicians and endocrinologists have significant concerns regarding the negative impact of prolonged systemic estrogen treatment on cancer risk, fertility, and mood (62, 63). Albumin's abundance, long circulation half-life, and ability to transport various substances make it an effective carrier for LN-targeted drug delivery (64). Its biocompatibility and lymphatic affinity enhance the delivery of therapeutic agents to LNs, improving outcomes in diseases involving LNs. We leverage its lymphatic transport by binding drugs to albumin, offering more efficient and targeted therapies for metastatic cancers and autoimmune disorders. Therefore, this study rationalizes developing novel precision therapeutics which is exemestane-loaded A-NP specifically targeting LNs, to treat MS and other autoimmune disorders that can be managed explicitly with immunosuppressive estrogens in LNs. Our results suggest that targeting LN has the potential to overcome the side effects of systemic exogenous estrogen.

The significance of estrogen synthesis in the LNs during aging has not been explored. Here, we show that middle-aged male mice, not female mice, have lower 17β-estradiol levels in the LNs, possibly due to decreased aromatase expression than younger mice, and exhibit nonremitting and IFN-β-resistant EAE disease. Incidentally, middle-aged male patients with MS tend to develop a progressive form of MS, which is nonremitting, IFN-β insensitive, and associated with more significant CNS neuronal damage than relapsing–remitting MS (65, 66). This suggests that lower LN estradiol levels in middle-aged men may contribute to these observations.

Interestingly, the reduction of estrogens in LNs is independent of levels of gonadal estrogens, suggesting a unique synthetic machinery. Future investigations may focus on elucidating this machinery and the mechanisms by which sex-dependent aging diminishes LN estrogen activity. In addition, although it is not easy to collect normal human LN samples, we will evaluate the expression level of aromatase in male and female humans at different age points.

Evaluating the therapeutic efficacy of LN-specific E2 agonists after disease onset or peak time is essential for determining their clinical relevance. While this aspect was not within the scope of the current study, it is a critical consideration for developing translational strategies for MS treatment. Future studies should aim to investigate the administration of LN-specific E2 agonists just after starting the symptoms or at peak time. These studies would provide key insights into the potential of these targeted therapies to induce disease remission, reduce neuroinflammation, and promote neuroprotection during established disease phases. Addressing this gap would significantly enhance the translational potential of LN-specific estrogen modulation in MS and related autoimmune conditions.

This study has some limitations that must be acknowledged. First, while the EAE mouse model is widely accepted for studying MS, it does not fully replicate human MS pathology. Key differences in immune system complexity, hormonal regulation, and CNS architecture limit the direct translatability of findings to human MS (67–69). Second, the study primarily relies on mouse data, with limited validation in human samples. Although aromatase expression was detected in human cervical LNs, the restricted availability of human LN tissue samples constrains the translational relevance of the findings. Third, we determined the relationship between estrogen decline and EAE progression in middle-aged mice, which show LN-estrogen decline, particularly in males, but not gonadal estrogen. More longitudinal analyses could strengthen the claims about age and sex differences in MS progression. The optimal timing for this study must be carefully determined, as aging also affects gonadal estrogen levels. A decline in gonadal estrogen levels complicates the evaluation of LN-derived estrogen.

In conclusion, our studies suggest that 17β-estradiol level and/or aromatase expression levels in human LNs could be a biomarker for the IFN-β-resistant MS phenotype. In addition, the findings indicate that an LN-specific treatment with 17β-estradiol, ERα agonists, and ERα's downstream signals (24) may be considered a therapeutic approach for treating middle-aged patients with MS and patients with progressive MS that has nonremitting IFN-β-resistant, or severe gray matter atrophy phenotypes. Such a targeted therapeutic approach may reduce or eliminate the inevitable side effects of systemic treatment regimens.

## Materials and methods

### Animals

Eight-week-old healthy WT male and female mice of the C57BL/6 were purchased from Jackson Laboratories. Cyp19-RFP mice were generated by crossing Cyp19-cre mice with RFP (Tomato)-floxed mice. All the mice were group-housed (3–5 mice per cage) in a specific pathogen-free facility with a 12-h light–dark cycle in the Veterinary Medicine Basic Sciences Building at the University of Illinois at Urbana-Champaign, Urbana, IL, USA. Diet and water were provided ad libitum. All mouse experiments were approved by the University of Illinois Animal Care and Usage Committee (IACUC) under protocol number 22140.

### Reagents

Recombinant IFN-β used in EAE treatment is human recombinant IFN-β-1b (Betaseron, Bayer HealthCare Pharmaceuticals), as previously reported (39, 40). MOG35–55 peptide was synthesized by United Peptides. Heat-killed *Mycobacterium tuberculosis* was purchased from BD Difco. Pertussis toxin (181) was purchased from List Biological. An enzyme-linked immunosorbent assay (ELISA) kit for detecting 17β-estradiol was purchased from DRG International Inc. Aromatase (PA1-21398) and MECA-79 (sc-19602) antibodies were purchased from Invitrogen and Santa Cruz Biotechnology, respectively.

### Ex vivo imaging of endogenous fluorescence

Cyp19-RFP mice were euthanized using CO_2_ overdose. Mice were dissected to expose internal organs. Distribution of RFP fluorescence was acquired using In-Vivo Xtreme II (Bruker). The image data were processed to apply spectral corrections, ensuring accurate fluorescence intensity measurements.

#### Immunohistochemical localization of aromatase and MECA-79 in mouse LNs

Collected LNs were fixed in 4% paraformaldehyde, then transferred to 20% sucrose for a minimum of 15 h, cryosectioned into 30-μm thickness with a cryostat, and mounted onto poly-L-lysine-coated slides. After overnight drying, samples were permeabilized with 0.5% Triton-X for 15 min, washed with 0.05% Tween-20/Tris-buffered saline (TBS) solution, and then blocked with 2% FBS solution. Samples were then incubated overnight at 4 °C with the primary antibodies, biotin anti-MECA-79 (1:200) (BioLegend, 120804) and/or polyclonal rabbit antiaromatase (1:50) (Cell Signaling, 14528S). Then, samples were washed and incubated with a species-specific secondary antibody: streptavidin Alexa-488 (BioLegend, 405235) and donkey antirabbit A568 (Fisher Scientific, A10042). Slides were rewashed before they were left to dry and coverslipped with ProLong Gold Antifade reagent application (BioLegend, P36930). Images were captured using the Nikon A1 confocal systems with 10× and 20× objectives.

#### Immunohistochemical localization of aromatase in human LNs

The experimental protocol was approved by the institutional review board at Akita University Hospital (permit number: 2422). Normal cervical LN samples were isolated from patients with benign thyroid diseases such as Graves’ disease and follicular adenoma. Written informed consent for using samples was obtained from all patients at each hospital. The methods in this study were carried out under the approved guidelines. Resected human LNs were determined to be normal by pathological diagnosis. LN tissues were fixed in neutral buffered formalin for 24 h, processed routinely, embedded in paraffin, sectioned at 5-µm thickness, and deparaffinized in xylene. Tissue sections were blocked in bovine serum albumin (3%) in TBS with 0.5% Triton X-100 for 1 h. The slides were incubated overnight with the aromatase and anti-MECA-79 antibodies (1:500 dilution) at 4 °C. Then, the slides were washed with TBS before they were incubated with the secondary antibodies, donkey antirat IgG (H + L) highly cross-adsorbed secondary antibody, Alexa Fluor 488, Invitrogen A-21208, and donkey antirabbit IgG (H + L) highly cross-adsorbed secondary antibody, Alexa Fluor 568, Invitrogen A10042, at 1:500 dilution for 2 h at room temperature. Then, the slides were washed and incubated with DAPI at a dilution of 1:10,000 for 3 min before they were coverslipped with ProLong Gold Antifade reagent application (BioLegend, P36930). Images were captured using the Nikon A1 confocal systems and analyzed using ImageJ software.

#### Induction of active EAE

In 6- to 8-week-old mice, EAE was induced by s.c. injection of myelin oligodendrocyte glycoprotein 35–55 peptide (100 µg/mouse) emulsified in complete Freund's adjuvant including heat-killed *Mycobacterium tuberculosis* (200 µg/mouse) on day 0, along with intraperitoneal (i.p.) injection of pertussis toxin (200 µg/mouse) on days 0 and 2. EAE score is defined as follows: 0.5 for partial tail limpness; 1 for tail limpness; 1.5 for reversible impaired righting reflex; 2 for impaired righting reflex; 2.5 for paralysis of one hind limb; 3 for paralysis of both hind limbs; 3.5 for paralysis of both hind limbs and one fore limb; 4 for paralysis of hind and fore limbs; and 5 for death.

#### IFN-β injection

As in a previous study (70), mice were i.p. injected with IFN-β (47 μg/kg) every other day from day 0 to 10 post-EAE induction.

### Golgi-Cox staining, imaging, and analysis

At chronic- and late-phase disease (33-day post-EAE induction (dpi)), animals were perfused with 4% paraformaldehyde (71) with no post-fixing and then processed according to the FD Rapid Golgi staining kit protocol (FD Neurotechnologies, #PK401A), which involves 17 days of incubation in metallic solution in the dark at RT. Spinal cords were frozen in Tissue-Tek OCT compound (Sakura Finetek) and stored at −80 °C until sectioning. Spinal cords were transversely cut into 50-µm sections using a cryostat (Thermo Scientific CryoStar NX50) and mounted onto poly-L-lysine-coated glass slides. At least eight sections were developed for Golgi-Cox neuronal staining performed according to the manufacturer's protocol. After drying, slides were covered with resinous promount and 0.17-μm coverslips.

### Sholl analysis

Microscopic images for analysis were captured using a 40× objective lens (Leica DM2000). Twenty individual neurons in the lumbar spinal cord ventral horn regions in each animal were selected for Sholl analysis. The software ImageJ was used for quantification. Neuronal soma was selected manually using the “polygon selection” tool to remove background signals and signals derived from cells other than the chosen neuronal soma (44). To initiate the Sholl analysis plugin, the image was converted into 8-bit, and a standard threshold was applied. Distance between rings was set at 10 microns with the end radius being at 200 µm (44, 45).

### A-NPs preparation and treatment

Nanoparticle was prepared using the nanoprecipitation method. PLA solution (50 mg/mL in DMF, 1 mL), exemestane solution (10 mg/mL in DMF, 0.225 mL), and DMF (1 mL) were mixed and added dropwise into a glass vial prefilled with BSA aqueous solution (10 mL, 2.5 mg/mL) under vigorous stirring. For control NP, no exemestane solution was added. The resulting emulsion was dialyzed against DI water for 48 h, with the water changed every 8 h (MWCO = 3.5 kDa) and then lyophilized. Exemestane-loaded A-NPs were prepared in PBS at a 7.5-μg/mL concentration. A 200-μL volume of the solution was administered to mice via s.c. injection every other day, starting 4 days prior to and continuing until 8 days post-EAE induction.

### Enzyme-linked immunosorbent assay

We used an ELISA kit to detect 17β-estradiol (DRG International Inc, EIA4174) and performed the ELISA according to the manufacturer's protocol.

### Western blotting analysis

LN tissues were lysed with RIPA lysis buffer (Thermo Scientific, 89901) supplemented with a protease inhibitor cocktail (Thermo Scientific), and the concentration of total protein was determined using the BCA Protein Assay Kit (Pierce BCA Protein Assay Kits, Thermo Scientific, 23227). Fifty μg of protein was separated by sodium dodecyl sulfate–polyacrylamide gel electrophoresis– and transferred onto PVDF membranes (Millipore), followed by incubating with BSA 3% in TBST for 2 h at room temperature. The membranes were incubated with specific primary antibodies (rabbit antiaromatase polyclonal antibody, Invitrogen, 21398, 1:250) at 4 °C overnight. Glyceraldehyde-3-phosphate dehydrogenase (GAPDH) is used as a loading control to normalize protein levels. The membranes were further washed and incubated with a secondary antibody, horseradish peroxidase -conjugated anti-rabbit secondary antibody (MyBioSource, MBS440123, 1:2,500), at room temperature for 1 h. Chemiluminescence was detected using an ECL Immobilon Western kit (SuperSignal West Femto Maximum Sensitivity Substrate [Thermo Scientific, 34094]), followed by exposure in FluorChem R System Bio-Techne, which includes ProteinSimple (92-15313-00). Quantification of images was performed by ImageJ.

### Statistical analysis

Statistical analysis of all results was evaluated with two-tailed unpaired Student's t tests and *P*-values. The criterion of significance was set as *P* < 0.05. Animals were randomly used for experiments under the criteria aforementioned in the section “Animals.” All behavior experiments were performed in a blinded and randomized fashion. No animals or data points were excluded. Quantifications were performed from at least two experimental groups in a blinded fashion. Measurements were taken from distinct samples for each experiment. For each test, the experimental unit was an individual animal. No statistical methods were used to predetermine sample sizes, but our sample sizes are similar to those generally employed in the field (25, 39). Data distribution was assumed normal, but this was not formally tested. Statistical analyses and graphical presentations were computed with GraphPad Prism software (GraphPad, La Jolla, United States). According to previous reports, data significance in Sholl analysis was considered only if more than two adjacent points showed *P*-values < 0.05 (45). All bar graphs are presented as the mean ± SEM.

## Ethics statement

All animal experiments were conducted with the approval of the University of IACUC under protocol number 22140. The human study protocol was approved by the institutional review board at Akita University Hospital (permit number: 2422). Normal cervical LN samples were obtained from patients with benign thyroid conditions, including Graves’ disease and follicular adenoma. Written informed consent for sample collection and use was obtained from all participants at each respective hospital.

## Data Availability

Raw data are also available as supplemental files.
